# Genome-Wide Transcriptomic Analysis Reveals Insights into the Response to *Citrus bark cracking viroid* (CBCVd) in Hop (*Humulus lupulus* L.)

**DOI:** 10.3390/v10100570

**Published:** 2018-10-18

**Authors:** Ajay Kumar Mishra, Atul Kumar, Deepti Mishra, Vishnu Sukumari Nath, Jernej Jakše, Tomáš Kocábek, Uday Kumar Killi, Filis Morina, Jaroslav Matoušek

**Affiliations:** 1 Biology Centre of the Czech Academy of Sciences, Department of Molecular Genetics, Institute of Plant Molecular Biology, Branišovská 31, 370 05 Ceske Budejovice, Czech Republic; ajaymishra24@umbr.cas.cz (A.K.M.); atul.kumar@umbr.cas.cz (A.K.); deepti.mishra@umbr.cas.cz (D.M.); sukumari.nath@umbr.cas.cz (V.S.N.); kocabek@umbr.cas.cz (T.K.); killi@umbr.cas.cz (U.K.K.); 2 Department of Agronomy, Biotechnical Faculty, University of Ljubljana, Jamnikarjeva 101, SI-1000 Ljubljana, Slovenia; Jernej.Jakse@bf.uni-lj.si; 3 Biology Centre of the Czech Academy of Sciences, Department of Plant Biophysics and Biochemistry, Institute of Plant Molecular Biology, Branišovská 31, 370 05 Ceske Budejovice, Czech Republic; morina@umbr.cas.cz

**Keywords:** *Citrus bark cracking viroid*, differentially expressed genes, hop, pathogen, transcriptome analysis, viroids

## Abstract

Viroids are smallest known pathogen that consist of non-capsidated, single-stranded non-coding RNA replicons and they exploits host factors for their replication and propagation. The severe stunting disease caused by *Citrus bark cracking viroid* (CBCVd) is a serious threat, which spreads rapidly within hop gardens. In this study, we employed comprehensive transcriptome analyses to dissect host-viroid interactions and identify gene expression changes that are associated with disease development in hop. Our analysis revealed that CBCVd-infection resulted in the massive modulation of activity of over 2000 genes. Expression of genes associated with plant immune responses (protein kinase and mitogen-activated protein kinase), hypersensitive responses, phytohormone signaling pathways, photosynthesis, pigment metabolism, protein metabolism, sugar metabolism, and modification, and others were altered, which could be attributed to systemic symptom development upon CBCVd-infection in hop. In addition, genes encoding RNA-dependent RNA polymerase, pathogenesis-related protein, chitinase, as well as those related to basal defense responses were up-regulated. The expression levels of several genes identified from RNA sequencing analysis were confirmed by qRT-PCR. Our systematic comprehensive CBCVd-responsive transcriptome analysis provides a better understanding and insights into complex viroid-hop plant interaction. This information will assist further in the development of future measures for the prevention of CBCVd spread in hop fields.

## 1. Introduction

Viroids are small, single-stranded, circular, highly structured, non-protein-coding infectious RNAs with genomes ranging in size from 250 to 401 nt [1]. The viroid genome is approximately tenfold smaller than the smallest RNA virus and they solely depend on their own RNA, host factors, and host enzymatic machinery for autonomous replication and movement [2,3]. In addition, viroids have intrinsic potential for high rates of per-base *in vivo* mutation among all the nucleic acid-based pathogens, which make them alluring models to study structure-function relationship in RNA [4]. They are cosmopolitan in distribution and are the etiologic agents of diverse diseases affecting monocots and dicots, herbaceous and woody, and agronomic and ornamental plants [5]. Viroid-induced symptoms range from necrosis to less severe developmental disorders, including leaf chlorosis, stunting, flowering alterations, and fruit and seed deformations [2]. Currently, the International Committee on Taxonomy of Viruses (ICTV) classified 32 viroid species, whereas the NCBI Taxonomy Database includes 45 viroid species [6]. Phylogenetically, viroids are broadly classified into two families based on their mode and site of replication, the presence/absence of a hammerhead ribozyme, and structural properties namely, the *Pospiviroidae* and the *Avsunviroidae* [7]. The most abundant viroids in the family *Pospiviroidae*, replicate in the nucleus, consist of rod-like secondary structures and follow an asymmetric rolling-circle mechanism of replication, whereas members of the family *Avsunviroidae* replicate (and accumulate) in the chloroplast with a rod-shape, branched structure, exhibit ribozyme-like self-cleavage activity, and follow a rolling-circle mechanism of replication [2,8]. The viroid-specific small RNAs (sRNAs) are involved in post transcriptional gene silencing (PTGS), or RNA silencing of host mRNAs to induce disease symptoms in plants [9,10,11].

Hop (*Humulus lupulus* L., Cannabaceae) is a dioecious, twining perennial flowering plant, native to Europe, western Asia and North America. The lupulin glands, which are glandular trichomes that are present on hop cones (high density) and leaves (low density) [12] are composed of biosynthetic cells that secret a specific complex metabolome consisting mainly of terpenophenolics (hop bitter acids and prenylflavonoids) and terpenoids (essential oil components), which serve as an essential ingredient in the beer industry, contributing to the distinctive bitterness, flavour, aroma, and preservative activity [13,14]. In addition, the hop plant has been traditionally acclaimed for several therapeutic benefits, such as relaxation and sleep inducer, anti-inflammatory effect, estrogenic effect, antioxidant activity, and anti-tumor property [15]. The hop plant is infected by several viroids during its growth and development such as *Hop stunt viroid* (HSVd) and *Apple fruit crinkle viroid* (AFCVd) [16], *Hop latent viroid* (HLVd), and the recently reported *Citrus bark cracking viroid* (CBCVd) member of the genus *Cocadviroid* in the family Pospiviroidae. [17,18]. Among them, the CBCVd infection is the most aggressive causing dramatic morphological and anatomical changes, which include leaf epinasty, yellowing, premature flowering, and a reduction in cone size, dry root rotting, stunted growth, and dieback [18].

The comprehensive analysis of gene expression patterns in viroid-infected plant is crucial to understanding the cellular and molecular mechanisms for viroid pathogenesis and further development of disease management strategies. Previously published studies have used different methods, such as differential display reverse transcriptase PCR (DDRT-PCR) [19] and microarray analysis [20,21], to gain an overview of altered gene expression in host plant upon viroid infection. Recently, next-generation sequencing (NGS) technologies, such as Solexa/Illumina RNA-Seq and digital gene expression (DGE), have provided a novel and powerful platform for global profiling of transcriptome and have several advantages over microarray analysis in terms of sensitivity of range of detection, higher reproducibility, and cost efficiency [22]. However, limited studies have described the transcriptome profiling of plant-viroid interactions, which include the transcriptome profiling of *Potato spindle tuber viroid* (PSTVd)-infected tomato [23] and potato [24], *Peach latent mosaic viroid* (PLMVd)-infected peach [25], HSVd-infected hop [26], and cucumber [27]. These studies showed that genes involved in plant immune responses, protein metabolism, secondary metabolism, hormone signaling pathways, and cell wall structure were strongly up-regulated upon viroid-infection and thus provide new insights into host response against viroid pathogenesis. In this context, comparable transcriptome analyses of other viroid-plant interaction could resolve the molecular mechanism related to symptom development, developmental changes, and biological processes response, etc.

In our previous study, we provided the first comprehensive functional assessment of sRNAs (miRNAs) and their regulation in the response to *Citrus bark cracking viroid* (CBCVd) infection in hop [18]. Our results indicated that the CBCVd-infection in hop results in the significant differential modulation of miRNAs that are involved in several hormone pathways and transcriptional factors involved in the regulation of metabolism, growth and development [18]. This observation directed our research towards uncovering the transcriptional reprogramming associated with CBCVd pathogenesis in hop. In the present study, we employed a high throughput transcriptome sequencing approach to gain a comprehensive understanding of the global alteration in gene expression resulting from CBCVd-infection in hop, which would further facilitate the development of effective measurements against viroid diseases.

## 2. Materials and Methods

### 2.1. Preparation of Dimeric CBCVd Construct and Inoculation of the Hop Plants

The full-length monomeric cDNA of CBCVd was amplified from total RNAs isolated from CBCVd-infected leaves of the Slovenian hop cultivar “Celeia” by reverse transcription-polymerase chain reaction (RT-PCR) using specific primers (Table S1). The purified RT-PCR product was cloned into T-Vectors (Takara Bio Inc., Shiga, Japan) and it was confirmed by sequencing. The dimeric construct was generated by digesting *Sac*I termini of monomeric positive cDNA strand of CBCVd and cloning into the *Sac*I site of pBlueScript KS (+) vector (Stratagene, San Diego, CA, USA). For 35S::CBCVd construct, the dimeric cDNA of CBCVd was subcloned into the pLV07 binary vector via intermediate vector pLV68 harboring a 35S expression cassette as described previously [28]. The CBCVd cDNA was immobilized on microcarrier gold particles (1 μm) using a calcium-mediated precipitation protocol [29] and biolistically inoculated on three-month-old hop leaves (cv. Osvald’s 72) grown in a container (10 cm height with at least three shoots-stage) under natural light condition. The individual hop plant was inoculated five times with 250 ng DNA per viroid species and was placed in darkness for 24 h after covering with plastic bags. Subsequently, the mock and CBCVd-inoculated plants were grown under natural condition and inspected visually for symptoms development.

### 2.2. RNA Isolation, Detection and Quantification of Genomic RNA of CBCVd

Systemically infected and mock-inoculated leaves were harvested at 120 and 412 days post inoculation (dpi) from the shoot apex after appearance and assessment of disease symptom in CBCVd-infected hop plants. The high-quality total RNAs were extracted using Concert™ Plant RNA Purification Reagent (Invitrogen, Thermo Fisher Scientific, Waltham, MA, USA), followed by RNA purification and DNA contamination removal using DNA-free^TM^ DNA Removal kit (Ambion, Thermo Fisher Scientific, Waltham, MA, USA), according to the manufacturer’s instruction. The concentration of total RNA samples was measured at 260 nm absorbance using NanoDrop 2000 spectrophotometer (Thermo Fisher Scientific, Waltham, MA, USA), whereas the integrity of RNA samples used for cDNA libraries construction was confirmed by Agilent Technologies 2100 bioanalyzer (Agilent Technologies, Colorado Springs, CO, USA).

The hop plant samples were examined for the CBCVd-infection using RT-PCR and dot blot assay. RT-PCR amplification for CBCVd detection was performed using an One-Step RT-PCR Kit (Qiagen, Valencia, CA, USA). Three microliters of RNA sample (adjusted to a concentration of 0.1 μg μL^−1^) was added to master mixture buffer containing 4 μL 5× One-Step RT-PCR Buffer, 0.6 μL (20 μM) of each CBCVd-specific forward and reverse primer (Table S1), 0.8 μL dNTP mix (10 mM each dNTP), 0.8 μL of One-Step RT-PCR Enzyme Mix, and 10.2 μL RNase-free water. Reaction mixtures were incubated in a PE9700 thermal cycler (Bio-Rad, Richmond, CA, USA) using the following reaction condition: 50 °C for 30 min for the reverse transcription step, followed by a PCR amplification step with initial denaturation at 95 °C for 15 min and 40 cycles of cDNA melting at 94 °C for 15 s, primer annealing at 58 °C for 30 s, primer extension at 68 °C for 45 s, accompanied with final primer extension at 68 °C for 7 min. The amplification of PCR products was confirmed by agarose gel electrophoresis.

Real time quantitative PCR (qRT-PCR) was performed to determine (+) and (−) vd-sRNAs accumulation of CBCVd in hop plants by following two steps to overcome challenges that are associated with highly stable viroid RNA secondary structure [30]. In the first step, 1 μg of total RNA was mixed with 20 μM of either CVdCS_PS or CVdCS_MS primer (Table S1) to make volume 13.4 μL. The mixture was denatured at 95 °C for 3 min and immediately chilled in ice. Subsequently, components of One-Step RT-PCR Kit (Qiagen, USA) were added to make final volume of reaction mixture to 20 μL and reverse transcription was performed, as mentioned above. In the second step, the quantification was performed using 3 μL of 10× diluted RT samples, which was added to 20 μL reaction mixture containing 10 μL 2× SYBR Green Real-Time PCR Master Mix (Invitrogen, USA), 6 μL RNase-free water, 0.5 μL of each 10 μM forward (CVdQRT_F), and reverse (CVdQRT_R) primers. qRT-PCR cycles were performed on an IQ5 Multicolor Real-Time PCR Detection System (Bio-Rad, USA) with following temperature profile: 94 °C for 4 min, followed by 40 amplification cycles 94 °C for 20 s, 61 °C for 40 s, and 72 °C for 30 s. In addition, the melting curve program (heating rate of 0.1 °C per s and a continuous fluorescence measurement) was performed to examine the specificity of the amplified product. The 7SL RNA gene product was used as an internal reference gene for normalization of expression level [31]. All qRT-PCR experiments were performed in triplicate using the independent CBCVd-infected (CI) and mock-inoculated (MI) samples that are mentioned above. The quantification of vd-sRNAs of CBCVd and data analysis were performed in accordance with MIQE guidelines [32].

The dot blot analysis of CBCVd was performed with full-length CBCVd [α^32^P] UTP-labelled probes by following the previously described method [30].

### 2.3. Transcriptome Sequencing, Assembly and Differential Gene Expression Profiling

Total RNA from four CBCVd-infected (CI) and four mock-inoculated (MI) in equal quantities were used for RNA sequencing. Total RNA (5 μg) was pooled from each sample in same batch from three individual leaves and was used for cDNA synthesis using cDNA Synthesis System (Roche, Basel, Switzerland), as per the manufacturer’s protocol. The cDNA samples were sheared via nebulization into small fragments and they were used for library construction using Illumina TruSeq Stranded Total RNA Sample Prep with Ribo-Zero (plant) kit (Illumina, San Diego, CA, USA). The quality of library was evaluated using the Agilent 2100 Bioanalyzer system (Agilent Technologies, Santa Clara, CA, USA). The resulting libraries were then paired-end sequenced (2 × 100 bp) on an Illumina Hiseq™ 2500 platform (Illumia) using myGenomics (Atlanta, GA, USA) sequencing services. The paired-end RNA-seq reads were subjected to the removal of adapters, filtering of empty and low-quality reads, trimming of reads with ambiguous nucleotides based on PHRED quality scores (Q-score) using default parameters of Trimmomatic v0.30 program [33]. The trimmed reads shorter than 40 bp were dropped to eliminate the sequencing artifacts and the quality of reads were evaluated using FastQC tool [34]. The high-quality reads were *de novo* assembled using Trinity software package version v2.4.0 [35] with default parameters settings (K mer = 25) and termed as unigenes. The evaluation of assembly was performed using Bowtie2 aligner by mapping the filtered reads against unigenes. All assembled unigene sequences were compared with the hop transcriptome database of HopBase genomic resources repository (http://hopbase.org/) using MEGABLAST at typical cut-off *E*-value of 1.0 × 10^−5^, with similarity level and alignment length more than 95% and 100 bp, respectively. In order to calculate the expression level of each unigenes, the clean reads for eight experimental samples were mapped to the unigenes dataset and normalized to the number of fragments per kilobase of exon per million mapped fragments (FPKM) by expectation-maximization (RSEM) protocol using in-built scripts in the Trinity software package [36]. The obtained count value was exported to DESeq2 R package [37] for determining differentially expressed gene transcripts (DEG) using the Benjamini and Hochberg approach [38] for controlling the false discovery rate (FDR). The expression of unigenes with FDR adjusted *p*-value ≤0.05 and at least a two-fold change (≥2 or ≤−2) was considered significantly different between CI and MI-libraries. The gene expression values were imported from DESeq2-normalized FPKM data sets and matrix distance for expression heatmap was calculated using the Euclidean distance and complete-linkage methods. A heatmap was constructed using the R statistics package heatmap3 [39].

### 2.4. Functional Annotation and Gene Enrichment Analysis

The assembled unigenes were aligned against the NCBI non-redundant (nr) protein database of *Viridiplantae* using BLASTX with a significance cut-off *E*-value of 1.0 × 10^−5^. Blast homology searches and homology-based functional annotations were carried out using Blast2GO Command line tools (Version 1.4.1) (Biobam, Valencia, Spain) [40]. Gene Ontology (GO) terms comprising of three functional groups, such as biological processes, molecular functions, and cellular components were assigned to the unigenes by Blast2GO program. The bidirectional best hit (BBH) method was used for KEGG (The Kyoto Encyclopedia of Genes and Genomes) pathway assignment of the assembled sequences using the online KEGG Automatic Annotation Server (KAAS; http://www.genome.jp/kegg/kaas/) to gain an overview of the biological pathways. The DEGs associated GO terms were enriched with respect to the GO terms that are associated to non-differentially expressed genes. The Fisher statistical test was performed to find enrichment of functional categories with Bonferroni’s correction (FDR ≤ 0.05) using the AgriGO toolkit [41]. The cut off *p*-value less than 0.05 was used as qualifying parameter for GO terms enrichment analyses, which were visualized using ReviGO [42]. Similarly, KEGG metabolic pathway annotation and enrichment of the DEGs were performed using hypergeometric test equivalent to Benjamini and Hochberg’s correction method with 5% FDR. The pathway visualization of DEGs was performed using MapMan tool (Forschungszentrum Jülich, Germany) [43]. The Log_2_FC values of DEGs were assigned to functional categories (or bins) by Mercator (http://mapman.gabipd.org/web/guest/mercator). In the case of expression data for duplicated gene identifiers, the lower value of fold-change was used for the analysis to avoid an overestimation of the data. The logarithm values of gene expression values were used to construct the gene regulatory modules following the methods as described previously [44]. The ortholog group assignment between DEGs of hop and *Arabidopsis thaliana* were performed using OrthoMCL [45] and selected DEGs with *A. thaliana* ortholog were subsequently used for network construction using NetworkAnalyst [46].

### 2.5. Validation of Differentially Expressed Genes by qRT-PCR

To confirm the results of transcriptome data, 12 randomly selected candidate DEGs were subjected to quantitative real-time PCR (qRT-PCR) analysis using designed specific primers (Table S1). Aliquots of the total RNA (5 μg) extracted for sequencing from leaves of CI and MI hop plants was treated with DNase I (Ambion, USA) and reverse-transcribed to first strand cDNA using Superscript^®^ III First-strand cDNA Synthesis kit (Invitrogen, USA), according to the manufacturer’s instructions. PCR amplification was performed in an IQ5 Real-Time PCR Detection System (Bio-Rad, CA, USA) using 20 μL of reaction mixture containing 10-fold diluted cDNA, 10 μL SYBR Green Real-Time PCR Master Mix (Invitrogen, USA), 10 μM of forward and reverse gene-specific primers (Table S1) under following conditions 95  °C for 5 min, followed by 40 cycles at 95  °C for 15 s, 58  °C for 30 s, and 72 °C for 30 s. The relative expression levels (fold-change) of the selected genes were calculated by the comparative *C*_t_ (2^−ΔΔ*C*t^) method [47] and hop glyceraldehyde 3-phosphatedehydrogenase (GAPDH) gene was used to normalize the amount of template cDNA in each reaction as the internal control [48]. The fold change of each gene was determined by three independent biological replicates and based on that standard deviation was calculated.

## 3. Results

### 3.1. Biolistic Inoculation of Hop with cDNA of CBCVd and Infectivity Confirmation

**Figure 1:**
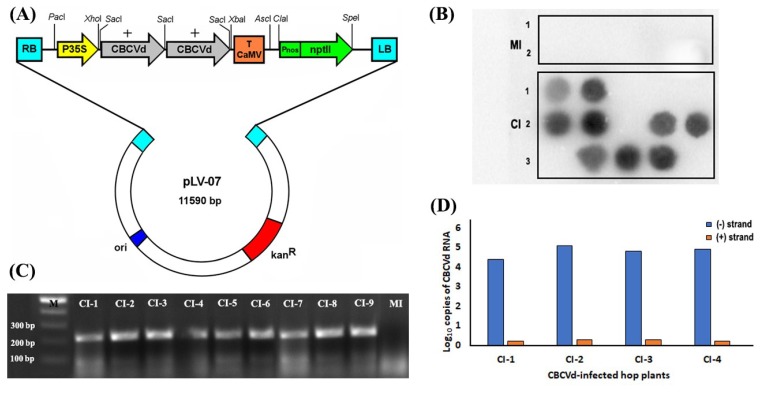
Construction of *Citrus bark cracking viroid* (CBCVd)-infectious construct and CBCVd detection and quantification: (**A**) Schematic diagram of a plasmid including the CBCVd (+) dimer created by cDNA cloning in *Sac*I restriction site. The CBCVd (+) dimer was re-cloned from pPCR-Script to *Xho*I–*Xba*I sites of intermediary vector pLV-68. Finally, modified expression cassette containing CaMV 35S promoter, viroid cDNA and CaMV terminator was cloned into *Pac*I and *Asc*I sites of the plasmid pLV-07. ori: origin of replication; kanR: kanamycin resistance gene; RB: left border of T-DNA; RB: right border of T-DNA; T CaMV: terminator from Cauliflower mosaic virus; Pnos: nopalin synthase promoter; nptII, Neomycin phosphotransferase II; (**B**) dot blot hybridization analysis of a [^32^P]-dCTP-labeled CBCVd cRNA probe to total nucleic acids isolated from mock inoculated (MI) and CBCVd-infected (CI) leaves of hop; (**C**) agarose gel electrophoresis analysis of mRT-PCR reaction for CBCVd-infected (CI-1 to CI-9) and mock inoculated (MI) leaves of hop after dormancy; (**D**) strand-specific real-time RT-qPCR analysis of reverse transcribed (+) or (−) CBCVd strands of CBCVd-infected hop plants after dormancy.

The hop plants were biolistically inoculated with infectious dimeric construct of cDNA of CBCVd (Figure 1) and examined for incidence of infection at pre-dormancy period (120 dpi) and after the appearance of typical symptom at post-dormancy period (412 dpi) following dot blot analysis, RT-PCR, and qRT-PCR (Figure 1). The positive hybridization signal was detected with minus strand-specific probe in nine out of fifteen CBCVd-inoculated plants at the pre-dormancy period (Figure 1B). The RT-PCR product of approximately 228 bp was observed in samples that were positive to CBCVd infection in dot blot analysis (Figure 1C). The strand-specific RT-qPCR (ssRT-qPCR) confirmed the result of minus strand-specific dot-blot hybridization and suggested the higher accumulation of minus multimeric strand as compared with plus polarity (Figure 1D). The nine CI hop plants were visually inspected for onset of typical symptoms such as stunted growth, leaf malformation, and bine cracking during the 14-months (Figure S1). After the dormancy period (14-month), dot-blot hybridization and ssRT-qPCR displayed the similar trend of excess of minus strand over plus strand in all nine symptomatic plants, which was corroborated with our previous studies suggesting that CBCVd utilizes minus strand as replicative intermediates [30].

### 3.2. Illumina Sequencing, De Novo Assembly and Functional Annotation of Unigenes

To compare the transcript profiles of hop in response to CBCVd-infection, total RNA was isolated from leaves of the apex of CI and MI prior to dormancy and post-dormancy of each individual plants and were pooled together in an equimolar amount to yield a final sample for sequencing with four biological replicates. Illumina RNA-Seq deep-sequencing run generated over 33.75 and 40.02 million raw reads in CI and MI libraries, respectively (Table 1). The removal of adapter sequences, shortest reads, ambiguous regions, and filtering out low-quality reads at high stringency using Trimmomatic software, resulted in retention of 24.39 and 36.68 million high-quality reads in CI and MI samples, respectively. The sequence abundance profiles of biological replicated samples showed high correlation (Table S2), indicating high quality reliable gene transcript data.

*De-novo* assembly with “Reduce” option to reduce redundancy in assembled unigenes and mapping reads back to contigs produced 27,094 unigenes from MI and CI combined libraries with length ranging from 90 to 2590. The N50, N75 value of *d*e-novo** assembly and average length were computed as 469 bp, 324 bp and 433 bp, respectively. The average unigene size of hop was longer than the average length of unigenes those identified in previous studied in *Camellia sinensis* (355 bp) [49], *Spartina alterniflora* (386 bp) [50], and *Eucalyptus grandis* (247 bp) [51]. The average GC content of assembled unigenes was 41.50% with normal distribution and were comparable to White clover (38.90%) [52], Spinach (42.50%) [53], *Sophora flavescens* (39.30%) [54].

**Figure 2:**
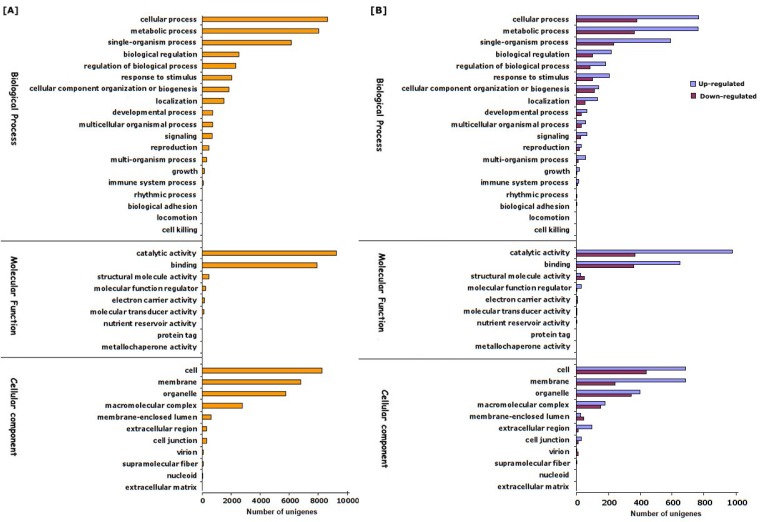
Gene Ontology (GO) terms assigned to the unigenes (**A**) and the differentially expressed genes (**B**).

In order to gain comprehensive functional descriptions, unigenes were first used for homology searching against NCBI nr protein databases with an *E*-value cutoff of 1.0 × 10^−5^, which showed that 21,869 (78.38%) assembled unigenes aligned to nr protein database, whereas the remaining 6035 (21.62%) did not show homology to any sequence in the database. The *E*-value distribution of the predicted proteins showed that more than 6.4% of the annotated unigenes showed *E*-values less than 1.0 × 10^−10^ and 51.64% of the mapped unigenes had significant hits with a stringent threshold of less than 1.0 × 10^−45^, confirming the consistency of annotated results (Figure S2A). The species distribution of unigenes based on their alignment against nr protein database showed that approximately 48.23% of total unigenes were matched with sequences from six top-hit species, namely, *Morus notabilis* (35.29%), *Ziziphus jujube* (12.47%), *Juglans regia* (3.72%), *Prunus persica* (2.66%), *P. avium* (2.40%), and *Malus domestica* (2.32%) (Figure S2B), suggesting the significant sequence conservation of unigenes of hop with other plant species. The sequence homology based on GO classification using Blast2GO tool assigned 16,291 unigenes into 39 subcategories under the three main GO categories (Figure 2A). A total of 78,819 GO functional assignments were obtained, among them, biological processes comprised the largest category (35,950, 45.61%), followed by cellular component (24,818, 31.48%) and molecular functions (18,051, 22.90%) (Figure 2A). Under the “biological process” category, the most of unigenes were functionally assigned into “cellular process” (23.96%), “metabolic process” (22.23%), and “single-organism process” (17.03%), whereas in “molecular function”, majority of unigenes were subcategorized into “catalytic activity” (51.19%), “binding” (43.78%), and “structural molecule activity” (2.47%). In “cellular component”, distribution of unigenes into “cell” (33.13%), “membrane” (27.36%), and “organelle” (23.01%) represented the major groups of unigenes. The alignment of unigenes to COGs database for orthologous genes resulted in the classification of unigenes into 23 functional categories with 4679 functional annotations due to multiple COG functions of some unigenes. Among the 24 functional categories, majority of unigenes were associated with “signal transduction mechanism” (10.23%) followed by “post translational modification, protein turnover, chaperone function” (9.31%), “carbohydrate transport and metabolism” (6.94%), and “transcription” (6.28%) (Figure S3A). In order to categorize gene functions in the context of biochemical pathways, the 8372 KEGG annotated unigenes were assigned into five different functional groups (Table 2). The majority of unigenes were annotated into the “metabolism”, with most of them involved in “carbohydrate metabolism” (5.45%), “amino acid metabolism” (3.14%), “lipid metabolism” (2.87%), “energy metabolism” (2.27%), “biosynthesis of other secondary metabolites” (1.69%), and other sub-categories. In the secondary metabolism categories, the predominantly represented subcategories were prenylflavonoids biosynthesis, flavonoid biosynthesis, sesquiterpenoid and triterpenoid biosynthesis, and many more. This observation was in corroboration with previous reports that sparsely distributed lupulin glands on adaxial surface of hop leaves can biosynthesize secondary metabolites at a detectable limit [12]. The genetic information processing group were strikingly represented by “translation” (3.16%), followed by “folding, sorting and degradation” (2.73%). In addition, 2385 unigenes were classified into “environmental information processing”, including “signal transduction”, “signaling and interaction molecules”, and “membrane transport” (Table 2).

### 3.3. Identification and Functional Classification of Differentially Expressed Genes

**Figure 3:**
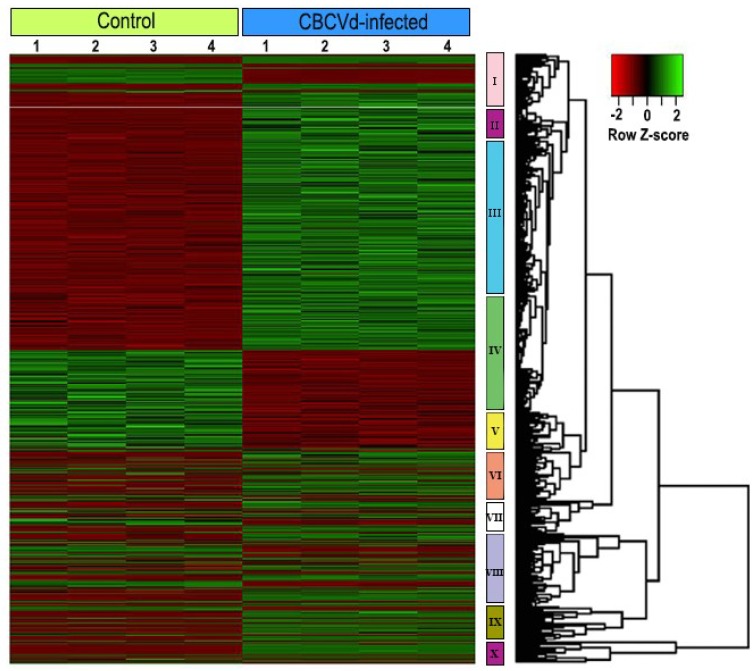
Heat map and complete linkage hierarchical clustering of log_2_ fold change of differentially expressed genes between CBCVd-infected compared with mock inoculated leaves of hop. Colors on vertical represent the clustered genes based on gene expression, the horizontal line represents the single gene. The color scale ranges from saturated red for log_2_ ratios −2.0 and below, to saturated green for log_2_ ratios +2.0 and above.

**Figure 4:**
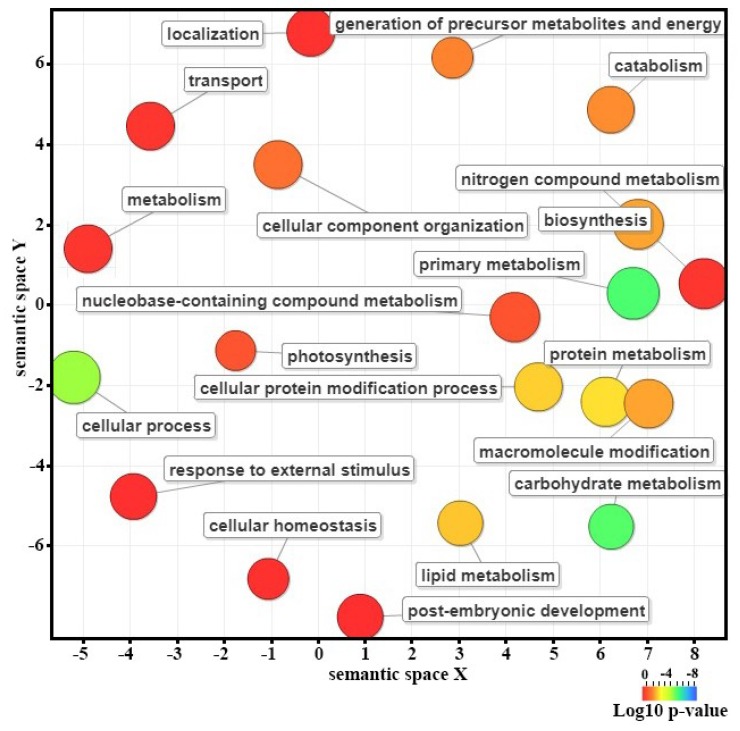
Gene ontology (GO) enrichment analysis of differentially expressed genes associated with CBCVd-infected hop plants. The GO terms cluster together in the semantic space according to functional similarity, without intrinsic meaning of semantic space units. Bubble color indicates indicate the *p*-value of enrichment according to the legend.

To identify the differentially expressed genes (DEGs) that are associated with CBCVd-infection in hop, gene expression levels in CI and MI samples were calculated using FPKM (expected number of Fragments Per Kilobase of transcript sequence per Million base pairs sequenced) [55]. The result of mapping of all reads utilizing non-redundant set of hop transcripts illustrated that the number of reads corresponding to each transcript ranged from 0.57 to 4716.55 (FPKM) in CI libraries, suggesting the extensive change of expression levels of hop transcripts (Table S3). The comparative transcriptome abundance analysis revealed significant differential expression level of 2209 unigenes (DEGs, *p* ≤ 0.05, logFC ≥ 2 or ≤−2), of which 1358 unigenes were up-regulated and 851 unigenes were found to be down-regulated in CI plants, which suggested the significant impact of CBCVd infection on host gene expression in leaves (Table S3). Approximately, 96.10% of DEGs (1297 up-regulated and 826 down-regulated) were annotated using BLASTx procedure against the nr-protein database of NCBI (Table S3). The hierarchical cluster analysis of the 2209 DEGs based on their FPKM values using the cluster distance method associated with complete linkage illustrated the relationship and degree of responses of DEGs in leaves of hop against CBCVd-infection (Figure 3). The candidate unigenes in clusters II, III, and IX were found to be up-regulated, whereas genes in clusters I and V were found to be significantly down-regulated in leaves of CI as compared to MI (Figure 3). Conversely, DEGs that are associated with cluster VII and VIII showed an intermediate pattern of expression. The annotation based on GO terms categorized 2209 DEGs into three classes: biological processes (19 terms, 45.42%), cellular function (10 terms, 31.37%), and molecular function (7 terms, 23.20%), whereas, 570 DEGs were not classified (Figure 2). Many of the DEGs are involved in cellular process, and catalytic activities. Furthermore, the GO enrichment analysis in this study provided the overview of statistically significant and relevant GO terms, which were altered as a result of CBCVd-infection in hop. A total of 32 GO terms were screened at *p*-value of 0.05 and statistically significant enriched GO terms, namely biological process, cellular component and molecular function was plotted in two-dimensional scatterplot in format of semantic representations of similar terms in closest coordinates (Figure 4). Among the various biological process categories, the GO terms linked to “metabolism”, “response to stimuli”, “cellular homeostasis” “transport”, etc. were significantly enriched (Figure 4). These pathways are associated with defense responses and are typically induced in plant-biotic interactions [56,57]. Similarly, in the molecular function category GO terms related to catalytic activity, such as “transferase activity, kinase activity” and binding activity, such as purine nucleoside binding, anion binding, nucleoside binding, adenyl nucleotide binding, and ATP binding were significantly enriched (Table 3), which plays a pivotal role in signal recognition and signal transduction during host-pathogen interaction. In addition, GO terms related to cellular components, such as thylakoid, plastid thylakoid membranes, and photosynthetic membranes were significantly enriched in the cellular component category.

The alignment of DEGs to COGs database for orthologous genes resulted in the classification of DEGs into 23 functional categories with 1987 functional annotations due to multiple COG functions of some DEGs (Figure S3B).

The mapping of DEGs in KEGG pathway assigned them to 7 KEGG pathway (Table 2). Several other disease-resistance pathways, including plant hormone signals, biosynthesis of secondary metabolites, amino acid, nitrogen, nucleotide, fatty acid, and sugar metabolism pathway were also found to be enriched (Table 2).

**Figure 5:**
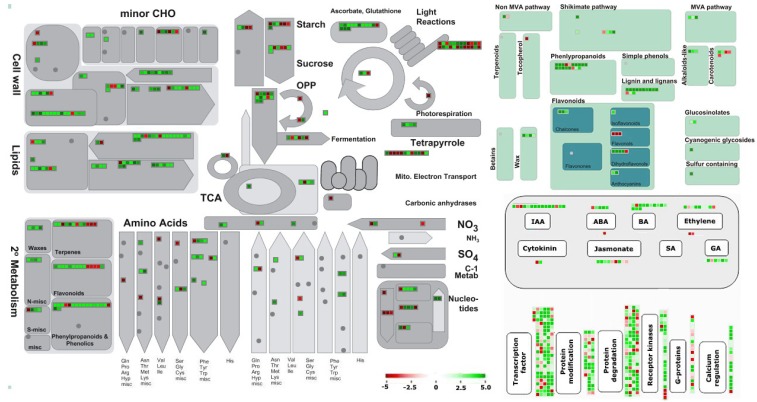
MapMan visualization of changes in transcript levels over the main metabolic process in CBCVd-infected compared with mock inoculated hop. The log_2_ fold changes of significantly differentially expressed genes were imported and visualized in MapMan. Red and green displayed signals represent a decrease and an increase in transcript abundance, respectively, in CBCVd-infected relative to the mock inoculated samples of hop. The scale used for coloration of the signals (log_2_ ratios) is presented.

Furthermore, DEGs were subjected to MapMan analysis to gain an unbiased, systematic overview of important biological functions and their coordinated response to CBCVd-infection in hop (Figure 5). Plants respond to pathogen attack by deploying an increased demand of energy and biosynthetic capacity, which is required for the priming of them for early defense [58]. As expected, genes that are involved in metabolism overview pathways, such as “cell wall”, “lipids”, and “secondary metabolic” pathways were consistently up-regulated. A cohort of genes associated with secondary metabolism pathway (phenlypropanoids, flavonoids lignin and glucosinolates) were highly expressed, which were the most significantly enriched pathways in the KEGG analysis. The down-regulation of genes that are involved in photosynthesis (e.g., PSI, PSII) and chlorophyll (tetrapyrrole) biosynthesis could be correlated to the appearance of disease symptoms, such as chlorosis on CBCVd-infected leaf tissues in hop. The expression of sucrose and starch biosynthesis genes was found to be diminished, which could be attributed to a reduction of photosynthesis activity. In addition, the hormone and signaling pathway related genes changed significantly.

The gene regulatory network analysis revealed potential regulatory elements of CBCVd-disease induction in hop (Figure S4). The identified hub genes might play crucial roles in regulating the innate immune response in hop against CBCVd-infection, but further experimental studies are required to validate the results.

### 3.4. Validation of Expression Pattern of Candidate DEGs by qRT-PCR

**Figure 6:**
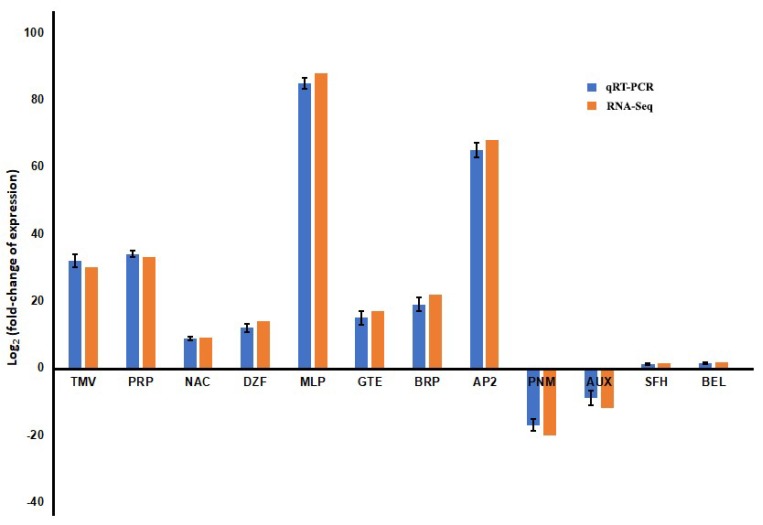
Quantitative real-time PCR (qRT-PCR) validation of differentially expressed genes from RNA sequencing. qRT-PCR analyses were normalized using GAPDH as an internal control gene. TMV: TMV resistance protein; PRP: Pathogenesis-related protein 1; NAC: NAC transcription factor 29; DZF: Dof zinc finger protein; MLP: MLP-like protein 423; GTE: GTE7-transcriptional factor; BRP: Brassinosteroid-regulated protein bru1; AP2: AP2-like ethylene-responsive transcription factor; PNM: Phosphomethylethanolamine *N*-methyltransferase; AUX: Auxin efflux carrier component; SFH: *S*-formylglutathione hydrolase; BEL: Homeobox protein BEL1. qRT-PCR analyses were normalized using *GAPDH* as an internal control gene. The fold change of each gene was calculated by the 2^−ΔΔ*C*t^ method.

In order to validate the results that were obtained from Illumina sequencing, twelve CBCVd-responsive annotated unigenes were selected randomly (based on expression patterns) and were subjected to qRT-PCR analysis using specific designed primers (Table S1). The result showed the consistent pattern of fold-changes of transcripts in qRT-PCR and RNA-Seq experiments (Figure 6). For example, both qRT-PCR and RNA-Seq analyses showed that genes encoding MLP-like protein 423, AP2-like ethylene-responsive transcription factor, and pathogenesis-related protein 1 were significantly higher and consistent in CI as compared to MI leaves of hop. The expression of genes encoding phosphomethylethanolamine *N*-methyltransferase and auxin efflux carrier component was found to be suppressed in qRT-PCR analysis, which was similar to the transcriptome data analysis. The expression pattern of two genes encoding *S*-formylglutathione hydrolase and Homeobox protein BEL1 were found not to be significantly up-regulated or down-regulated in CI leaves, which was in accordance with qRT-PCR expression patterns. Nonetheless, the expression patterns of genes by qRT-PCR were consistent with the DEGs analysis, indicating the reliable result.

## 4. Discussion

CBCVd has evolved a broad host range across host genotypes such as citrus, cucumber, tomato and eggplant etc. [59]. Recently, it has been discovered in hop, where it causes the most aggressive symptoms after first dormancy and complete dieback in 3–5 years [17]. Soon after the discovery of new host of CBCVd, quite intriguing issues that remain to be clarified about the transcriptional response of hop during the symptom development process. In this context, a previous study was mainly focused on the precise measurement of target transcript of CBCVd-derived vd-sRNAs by small/micro RNA target prediction computational tools [6]. To disentangle the CBCVd-hop interaction, in the current study, we investigated the systematic and comprehensive changes in the transcriptome of hop leaves following infection by the biolistically inoculated virulent variant of CBCVd.

Plants have evolved series of defense mechanism via signal transduction pathway to defend against pathogens. The plant innate immune system such as pattern-triggered immunity (PTI) and effector-triggered immunity (ETI) is conserved and constitute the first line of defense [60]. The PTI or ETI is triggered by the activation of various membrane-associated receptor-like kinases (immune receptor) upon the perception of non-self components of pathogenic origins via MAPK (mitogen-activated protein kinase) and CDPKs (calcium-dependent protein kinases), signalling cascades that act downstream of receptor complexes. The transducing signalling events eventually lead to transcriptional reprogramming, induction of the PR proteins, and hypersensitive response by the generation of reactive oxygen species (ROS) [61]. Our results revealed the extensive activation of signaling pathways genes (e.g., MAPK3 and CDPKs) as well as prominent marker genes that are associated with innate immunity (e.g., PR proteins, 1,3-beta-glucanase, and ROS biogenesis genes), which is in agreement with the results of a previous study [27]. Since viroids (CBCVd) lack the coding capacity, thus we cannot assume the effectors-based triggering of immune response in the host plant. Nevertheless, our DEGs data illustrated that the hop possesses the effective perception system, which can easily discriminate and recognise CBCVd infectious RNA. In the animal system, the double-stranded RNA (dsRNA)-activated protein kinase (PKR) binds to viral dsRNA and invoke innate antiviral responses [62]. The previous study focusing on the RNA-binding activities of viral and host proteins in plants suggested that the PKR homolog in plant P58^IPK^ plays an important role in viral pathogenesis. The silencing of P58^IPK^ caused massive cell death in *Nicotiana benthamiana* and *A. thaliana* plants upon infection with the tobacco mosaic virus (TMV) [63]. Intriguingly, the P58^IPK^ gene was not found to be differentially regulated in CBCVd-infected samples. Therefore, the role of P58^IPK^ in triggering innate immunity against CBCVd-infection in hop is uncertain. Nevertheless, among several elevated protein kinases in our data set (Table S3) yet-to-be-discovered candidate player might play the dominant role in binding and perception of CBCVd and the further activation of defense response.

In plant cell, chloroplast is a dynamic organelle, which is not only involved in photosynthesis, but also actively participates in the synthesis of numerous compounds, interorganelle signaling, and regulation of cell-death programs as a part of plant immune system [64]. Several chloroplasts associated proteins are transcribed by nuclear genes and viroid replication in the nucleus cause triggering of chloroplast-based signaling cascades resulted in the impairment of the structural (thylakoid membrane abnormalities and paucity of grana) and functional changes in the chloroplast that is associated with the process of photosynthesis [64,65]. Several studies using microarray and RNA-seq approaches revealed that viroid infection could modulate the efficiency of photosynthetic rate by down-regulating the chloroplast- and photosynthesis-related genes [20,23,27]. In this study, we observed that many genes that are associated with chlorophyll metabolism and photosynthesis pathway such as photosystem I subunit l, ferredoxin-oxidoreductase, 2-oxoglutarate, and Fe(II)-dependent oxygenase superfamily, glyceraldehyde-3-phosphate dehydrogenase, ferredoxin, etc. were down-regulated, which might be correlated to symptom development (chlorosis, stunting or mosaic) in CBCVd-infected hop plants.

Numerous elegant studies have demonstrated that plant hormones play prominent roles in the plant life cycle and act as an important regulator of metabolic pathways that are related to plant growth, development, and abiotic/biotic stress responses [66,67]. Viroids or viruses infections are frequently associated with phytohormone alterations, which causes the disruption of host cellular physiology resulting in the developmental disorders [23,68]. In our study, the expression level of several genes that are associated with hormone signal transduction pathways showed the altered expression. In general genes involved in indole-3-acetic acid (IAA), ethylene (ET), gibberellins (GA), jasmonate (JA), brassisteroid (BA), and abscisic acid (ABA) were up-regulated. Similarly, genes involved in IAA, ET, and BA-biosynthesis and responses were induced in PSTVd-infected tomato plants [69]. Salicylic acid (SA) has recently received attention in rendering basal resistance of tomato plants against *citrus exocortis viroid* (CEVd) [70]. However, changes of expression of genes that are involved in SA metabolism or response were not observed. A similar result was observed in PSTVd-infected tomato plants, which showed the altered transcript levels for several genes involved in GA biosynthesis and BR signaling, but not other genes that are associated with SA or JA dependent pathways [23].

Translational reprogramming is a critical component of the cellular response, which is required for rapid cellular adaptation under different types of stress conditions, including pathogen infection [27]. The pathogen utilizes host translation apparatus to their own replication and intercellular movement, and often equated with pathogen strategy to circumvent the mRNA competitiveness [27]. Intriguingly, the marked up-regulation of genes encoding ribosome biogenesis proteins were observed, suggesting that CBCVd enhanced the translation process of hop for their own repertoire. The mounting evidence indicates that viroids and viruses exploit or interfere the plant ubiquitin/proteasome system and heat shock proteins (HSPs) for biotrophic interactions [6,71,72]. In this context, previous in-depth studies illustrated that viruses can actively manipulate the ubiquitination machinery and/or the proteasome to promote their replication and movement [73]. In addition, viruses can direct the ubiquitin proteasome system to the modification of new targets, such as argonaute to enhance or suppress gene silencing in order to suppress innate immunity [72]. In our study, we found that several genes (28 genes) associated to protein degradation via ubiquitin pathway were upregulated as compared to the down-regulated genes (13 genes) in CBCVd-infected hop. Similarly, HSP (HSP70 and HSP90) family homologs were shown to be induced several folds in many plant interaction studies with RNA viruses and viroids [74,75]. It has been suggested that HSP chaperones are targeted by RNA viruses for replication, cell-to-cell movement, and regulation of host defense directly or indirectly through interactions with DnaJ [76]. It is interesting to note that several classes of HSP (HSP33, HSP70, HSP90) transcripts are induced to higher level in CBCVd-infected hop, reinforcing the role of HSPs during the CBCVd-infection cycle. Several lines of evidence indicate that transcriptional factors (TFs) play a pivotal role in the signal transduction process in plants against abiotic and biotic response [77]. Intriguingly, the gene expression level of major families of TFs, such as bZIP, WRKY, MYBs, MYC, bHLHs, AP2/EREBP, and ERFs were significantly altered in CBCVd-infected plants (Table S3), which is consistent with the results of previous study of PSTVd-infection in potato [24].

In our study, majority of genes involved in primary and secondary metabolism pathways were fluctuated in response to the CBCVd-infection. At the link between primary and secondary metabolism, phenylalanine ammonia-lyase (PAL) serves as first enzyme that is involved in biosynthesis of secondary metabolites, such as flavonoids, anthocyanins, prenylflavonoids, and bitter acids in hop [78]. Flavonoids constitute a large group of phenolic secondary metabolites and have been shown to have diverse biological functions, such as symbiotic nitrogen fixation, pollination and floral pigmentation, defense against different biotic and abiotic stresses, etc. [79]. Nonetheless, the bitter acids (humulone or α-acid and lupulone or β-acid) and prenylflavonoids (xanthohumol and desmethylxanthohumol) secondary metabolite constituents of hop is widely used in brewing industry as a source of flavour-active secondary metabolites [78]. Notably, several genes related to general flavonoids and prenylflavonoids were influenced by CBCVd-infection in hop. The genes involved in flavonoids biosynthesis pathway were up-regulated, which indicated the stimulation of defensive substances in hop upon CBCVd-infection. Similarly, the regulatory genes encode for TFs (*Hl*WRKY1 and *Hl*WDR5) involved in prenylflavonoids biosynthesis pathway were up-regulated, which was in agreement with a previous study [30]. In summary, the analysis of differential expression of CBCVd-infected hop will facilitate the investigations of the detailed mechanisms of plant responses to viroid infection and contribute to a better understanding of development of strategies to combat viroid diseases in hop production.

## Figures and Tables

**Table 1:** Statistics of RNA-seq analysis in mock-inoculated (MI), CBCVd-infected libraries (CI), and assembly for hop.

Item	Library	Number	Total Bases (GB)
Raw read	MI	33,752,449	3.21
	CI	40,023,124	3.60
Clean read	MI	24,399,800	1.79
	CI	36,686,240	2.72
Average Length (bp)	MI	421	
	CI	418	
**Unigenes**			
No. of Unigenes (n)		27,904	
Average Length (bp)		451	
Maximum Length (bp)		2590	
Minimum Length (bp)		90	

**Table 2:** Classification statistics for unigenes (UG) and differentially expressed genes (up-regulated (UR) and down-regulated genes (DR)) in CBCVd-infected hop plant according to Kyoto Encyclopedia of Genes and Genomes (KEGG) pathway analysis.

KEGG Categories	Number of			KEGG Categories	Number of		
	UG	UR	DR		UG	UR	DR
Metabolism				Organismal System			
Carbohydrate Metabolism	1457	168	54	Immune system	650	24	23
Energy metabolism	608	58	47	Endocrine system	692	66	29
Lipid metabolism	768	149	41	Circulatory system	93	8	3
Nucleotide metabolism	320	19	29	Digestive system	167	18	6
Amino acid metabolism	841	126	50	Excretory system	111	12	2
Metabolism of other amino acids	307	44	24	Nervous system	495	44	13
Glycan biosynthesis and metabolism	299	43	10	Sensory system	49	11	3
Metabolism of cofactors and vitamins	412	41	13	Development	74	5	6
Metabolism of terpenoids and polyketides	222	31	26	Aging	219	10	17
Biosynthesis of other secondary metabolites	453	110	10	Environmental adaptation	506	55	26
Xenobiotics biodegradation and metabolism	239	61	34				
Enzyme families	921	106	41				
**Genetic information processing**							
Transcription	316	71	60				
Translation	846	111	235				
Folding, sorting and degradation	730	201	65				
Replication and repair	271	63	60				
RNA family	0	0	0				
**Cellular Process**							
Transport and catabolism	916	174	52	**Unclassified**			
Cell growth and death	684	49	22	Metabolism	2007	44	11
Cellular community—eukaryotes	178	7	3	Genetic information processing	5896	6	5
Cellular community—prokaryotes	113	20	3	Cellular processes and signaling	1833	8	3
Cell motility	55	14	14	Viral protein family	0	0	0
**Environmental information processing**				Poorly characterized	585	8	6
Membrane transport	64	96	33				
Signal transduction	2134	165	63				
Signaling molecules and interaction	187	26	7				
				**Total**	26,718	2272	1149

**Table 3:** Gene ontology (GO) functional enrichment analysis of differentially expressed genes in CBCVd infected hop.

GO ID	Ontology	Category	Number of DEGs	Number of Unigenes in Subgroup	FDR	*p*-value
**GO:0008152**	metabolic process	P	1008	10,614	7.10 × 10^−92^	2.20 × 10^−94^
**GO:0044238**	primary metabolic process	P	816	8995	9.20 × 10^−59^	5.60 × 10^−61^
**GO:0005975**	carbohydrate metabolic process	P	199	866	5.00 × 10^−58^	4.60 × 10^−60^
**GO:0009987**	cellular process	P	949	11,684	2.70 × 10^−50^	3.30 × 10^−52^
**GO:0019538**	protein metabolic process	P	388	4009	3.80 × 10^−28^	5.80 × 10^−30^
**GO:0006464**	protein modification process	P	193	1474	3.70 × 10^−26^	6.70 × 10^−28^
**GO:0006629**	lipid metabolic process	P	135	841	3.40 × 10^−25^	7.30 × 10^−27^
**GO:0009058**	biosynthetic process	P	435	5118	2.50 × 10^−21^	6.80 × 10^−23^
**GO:0009056**	catabolic process	P	159	1307	1.00 × 10^−18^	3.10 × 10^−20^
**GO:0015979**	photosynthesis	P	34	162	5.80 × 10^−9^	2.30 × 10^−10^
**GO:0006810**	transport	P	136	1846	0.0025	0.00012
**GO:0051179**	localization	P	136	1922	9.90 × 10^−3^	0.00058
**GO:0019725**	cellular homeostasis	P	21	174	1.30 × 10^−2^	0.00082
**GO:0009605**	response to external stimulus	P	39	429	2.20 × 10^−2^	0.0014
**GO:0003824**	catalytic activity	F	1145	9638	3.60 × 10^−18^	4.10 × 10^−189^
**GO:0016740**	transferase activity	F	360	3321	6.90 × 10^−35^	1.60 × 10^−36^
**GO:0016787**	hydrolase activity	F	370	3468	8.10 × 10^−35^	2.80 × 10^−36^
**GO:0005488**	binding	F	859	11,258	1.50 × 10^−33^	8.60 × 10^−35^
**GO:0016301**	kinase activity	F	140	1641	1.20 × 10^−06^	8.50 × 10^−8^
**GO:0016772**	transferase activity, transferring phosphorus-containing groups	F	140	1887	7.70 × 10^−4^	7.10 × 10^−5^
**GO:0005215**	transporter activity	F	108	1477	7.10 × 10^−3^	0.00075
**GO:0008135**	translation factor activity, nucleic acid binding	F	21	181	1.10 × 10^−2^	0.0013
**GO:0005198**	structural molecule activity	F	54	659	1.40 × 10^−2^	0.0018
**GO:0016020**	membrane	C	426	4068	2.80 × 10^−38^	1.60 × 10^−40^
**GO:0005737**	cytoplasm	C	582	6822	1.30 × 10^−30^	2.10 × 10^−32^
**GO:0005623**	cell	C	1015	15,217	7.40 × 10^−20^	2.90 × 10^−21^
**GO:0043226**	organelle	C	594	8155	5.60 × 10^−16^	3.20 × 10^−17^
**GO:0005840**	ribosome	C	79	524	1.10 × 10^−13^	6.80 × 10^−15^
**GO:0009536**	plastid	C	227	2965	3.20 × 10^−7^	3.80 × 10^−8^
**GO:0005618**	cell wall	C	49	403	3.00 × 10^−6^	3.80 × 10^−7^
**GO:0005829**	cytosol	C	84	912	1.80 × 10^−5^	2.50 × 10^−6^
**GO:0005886**	plasma membrane	C	100	1456	0.041	0.0064

## Supplementary Materials

The following are available online at http://www.mdpi.com/1999-4915/10/10/570/s1, Figure S1: Symptoms induced in hop plants after 145 days of biolistic inoculation of CBCVd inoculum. An Infected plant showing stunted growth, smaller leaves with chlorosis compared to control. Figure S2: Non-redundant database classification of hop unigenes. (A) *E*-value distribution. (B) BLASTx top-hit species distribution. Figure S3: Histogram presentation of clusters of orthologous groups (COGs) classification of unigenes (A); and differentially expressed genes in CBCVd-infected compared with mock inoculated hop (B). Figure S4: The analysis of protein-protein interaction network of CBCVd-responsive differentially expressed genes (DEGs) in hop by STRING (edge value cutoff of 0.70). The lines indicate network-predicted interactions. The regulatory network shows the DEGs of hop homologous to *Arabidopsis thaliana*. Table S1: Primers used for RT-PCR detection and qRT-PCR analyses. Table S2: Correlation analysis of sequencing libraries. Table S3: The unigenes differentially expressed between CBCVd-infected and mock inoculated hop plants. The complete raw RNA-seq datasets for four biological replicates of mock-inoculated and CBCVd-infected hop plants have been deposited in the NCBI Sequence Read Archive under accession number SRR6206403 and SRR7904254, respectively.
